# Current Treatment Options and the Role of Functional Status Assessment in Classical Hodgkin Lymphoma in Older Adults: A Review

**DOI:** 10.3390/cancers15051515

**Published:** 2023-02-28

**Authors:** Vittorio Ruggero Zilioli, Cristina Muzi, Chiara Pagani, Emanuele Ravano, Erika Meli, Rosa Daffini, Erika Ravelli, Roberto Cairoli, Alessandro Re

**Affiliations:** 1 Division of Hematology, ASST Grande Ospedale Metropolitano Niguarda, 20162 Milan, Italy; 2 Division of Hematology, ASST Spedali Civili, 25123 Brescia, Italy

**Keywords:** Hodgkin lymphoma, elderly, comorbidity, functional status, geriatric assessment, brentuximab vedotin

## Abstract

**Simple Summary:**

Hodgkin lymphoma (cHL) in older adults is typically characterized by a prognosis that is markedly worse than that of young patients, due to both greater difficulty in achieving adequate disease control and higher treatment-related toxicity. Although rarely included in clinical trials, older cHL patients represent an unmet clinical challenge, with disease incidence expected to increase in Western countries due to the aging of the general population. This review covers the current clinical and therapeutic landscape of cHL in older patients and describes the useful tools for these patients’ care. Particular attention is given to the currently available first-line regimens and the need for a baseline assessment of patient fitness as a criterion for better treatment selection.

**Abstract:**

Along with the fact that classical Hodgkin lymphoma (cHL) in older adults is frequently considered biologically different from cHL in younger patients, its most distinctive feature is its dismal clinical outcome due to the decreased effectiveness and greater toxicity of therapies. Although strategies to mitigate specific toxicities (e.g., cardiological and pulmonary) have obtained some results, in general, reduced-intensity schemes, proposed as an alternative to ABVD, have proved to be less effective. The addition of brentuximab vedotin (BV) to AVD, especially in a sequential scheme, has demonstrated good efficacy. However, the problem of toxicity persists even with this new therapeutic combination, with comorbidities remaining an important prognostic factor. The adequate stratification of functional status is necessary to distinguish between those patients who will benefit from full treatment and those who will benefit from alternative strategies. A simplified geriatric assessment based on the determination of ADL (activity of daily living), IADL (instrumental ADL), and CIRS-G (Cumulative Illness Rating Scale—Geriatric) scores is an easy-to-use tool that permits adequate patient stratification. Other factors of considerable impact on functional status such as sarcopenia and immunosenescence are currently being studied. A fitness-based treatment choice would also be very useful for relapsed or refractory patients, a more frequent and challenging situation than that is found in young cHL patients.

## 1. Epidemiology and General Characteristics

Classical Hodgkin lymphoma (cHL) in older adults is seen in the second peak of the incidence of cHL. Although there is no shared definition of “older patient” in the cHL setting, most studies agree that a patient ≥60 years of age is “older”. If we consider only these patients, cHL in older adults is a rare disease, accounting for about 20% of all cases of cHL, with a median age at diagnosis of around 70 years [1,2,3,4].

Despite accounting for about one-fifth of all new diagnoses of cHL, older patients are under-represented in clinical trials, which typically include less than 5–10% of patients aged ≥60 years. This may be due to the “biological diversity” of cHL in older adults as well as to the difficulty of treating these patients, who have poor tolerance and a reduced response to first-line therapy [5]. Furthermore, this under-representation is very unlike what occurs in the setting of diffuse large B-cell non-Hodgkin lymphoma (DLBCL), for example.

## 2. Is cHL in Older Adults a Biologically Different Disease?

With regard to the biological diversity of the disease, many studies have observed the characteristics of the presentation of cHL in older patients that are very specific to this age group and that are more evident than those observed in younger patients. The most relevant clinical features at presentation are primarily related to the higher incidence of advanced stages [6,7] and to the corresponding reduced incidence of localized stages with mediastinal bulk [8]. In addition, many older patients present with systemic symptoms of disease [6,7] and elevated erythrocyte sedimentation rate (ESR) levels [8]. Finally, a greater proportion of older patients present with reduced performance status, although it is not easy to assess to what degree this parameter is linked to their more aggressive disease and/or to other age-related organ or functional impairments [8].

In terms of histology, some authors have observed a higher incidence of the mixed cellularity subtype (classic Hodgkin/Reed–Sternberg cells in a diffuse mixed inflammatory background), in some cases finding it even more frequently than nodular sclerosis, by far the most diagnosed subtype of cHL in younger patients [8,9].

From a cytogenetic point of view, older patients with cHL more frequently present with the overexpression of chromosomal region 9p24.1 due to either polysomy, copy gain, or amplification [10]. It is noteworthy that the 9p24.1 region contains both the PD-L1 and PD-L2 genes, resulting in the overexpression of both proteins in Hodgkin cells and their microenvironment, as PD ligands are the therapeutic targets of effective new drugs such as nivolumab or pembrolizumab.

From a biological point of view, many authors have reported more frequent Epstein–Barr virus (EBV) infection of Reed–Sternberg cells [9,11], identified mostly through in situ hybridization methods commonly implemented in clinical practice. It is not entirely clear whether latent EBV infection plays a primary oncogenic role in cHL, although some authors support it [12]. Nevertheless, the prognosis of patients with EBV-positive cHL appears to be worse than that of EBV-negative patients, mainly due to a reduced response to first-line therapy, which tends to translate into reduced survival [13].

In general, some clinical and biological features can be observed that point to cHL being more aggressive in older patients than in younger patients. However, it should be emphasized that this evidence is drawn mostly from retrospective or population-based studies and, more generally, refers to a therapeutic paradigm that is not entirely current. There is, therefore, a need for large prospective studies in this setting aimed at extensively evaluating the prognostic factors (both lymphoma-related and patient- and/or therapy-related) within the current therapeutic scenario.

## 3. The Main Problem: Worse Outcome Compared with That in Younger cHL Patients

The greatest problem in the treatment of cHL in older patients is that their outcome is considerably worse than that usually observed in younger patients. Certainly, the overall outcomes for patients with cHL lymphoma have improved over time, as better supportive care and, more recently, better salvage and even first-line therapies have become available. This improvement has also benefitted older cHL patients, with the possible exception of those aged > 80 years, as reported in some studies. However, even with this improvement, the prognosis of cHL in older patients remains markedly poorer than that of cHL in younger patients and considerably different from that of the general age-matched population [3,14].

The possible greater biological aggressiveness of the disease, as outlined in the previous section, may be one of the causes of this different trend. In fact, it has been well documented that older patients tend to have a lower response rate to first-line therapy and a greater tendency to relapse. However, other factors may explain this inferior outcome: a certain tendency towards therapeutic inertia, the greater interval between diagnosis and treatment, the use of reduced or less effective therapeutic regimens, the greater toxicity experienced by older patients with traditional therapy regimens, and the tendency to treat them in smaller or less experienced centers [5].

Of these factors, the most studied is therapy-related toxicity in older patients. Ever since ABVD became the paradigm of first-line treatment of cHL, it has been observed that this course of therapy is decidedly more toxic in this patient population. In fact, older patients have more marked hematological toxicity, not only in terms of a greater likelihood of anemia and thrombocytopenia, with a greater need for transfusion support, but above all of the chemo-induced neutropenia, especially febrile neutropenia. The use of granulocyte colony-stimulating factor (G-CSF) in this setting has certainly reduced the incidence of neutropenia, particularly febrile neutropenia. However, it has partly contributed to another important ABVD-related toxicity, that of bleomycin.

**Figure 1:**
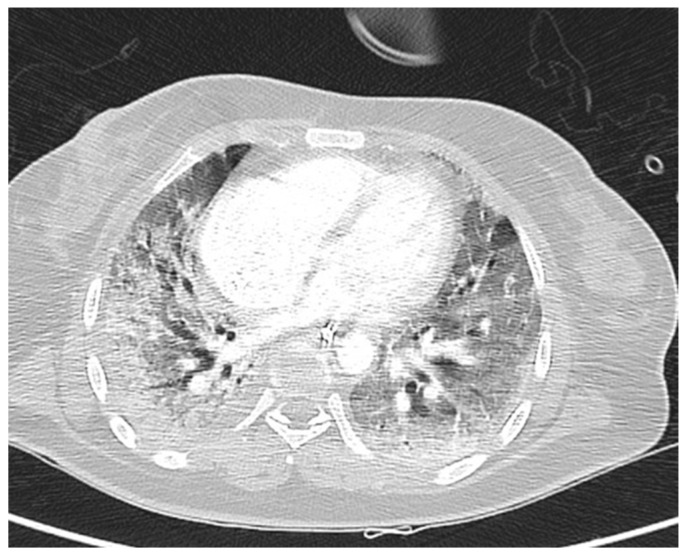
Bleomycin-related pulmonary toxicity: in this CT scan, bilateral severe fibrosis extended to all lung fields is represented. A 55-year-old woman experienced this fatal toxicity at the end of ABVD therapy for 6 cycles (with which she obtained CR).

The bleomycin toxicity that we are interested in analyzing here is pulmonary toxicity, which is known to take on different clinical features, from only a reduction in the alveolar–capillary diffusion of carbon monoxide to pneumonia and pulmonary fibrosis (Figure 1). Bleomycin toxicity is quite typical of the ABVD regimen, where it is much more frequently documented than other types of therapy. Several studies have documented an incidence of bleomycin toxicity in older patients that ranges from 5% to 35%, with an associated mortality of up to over 30% [15,16]. The most recognized risk factors for bleomycin toxicity are the use of G-CSF, the administration of more than two ABVD cycles, and, invariably, age >60 years. The association with cigarette smoking, however, appears milder and not always confirmed [17].

A third important toxicity in older patients is cardiological toxicity, certainly linked to the greater incidence of cardiological comorbidities (ischemic heart disease, arterial hypertension, and rhythm abnormalities) and/or risk factors (diabetes mellitus and dyslipidemia). The use of anthracycline in these patients is undoubtedly burdened by a greater risk of cardiovascular problems. However, it is well known that not only its use but also an adequate dose rate seem to be remarkably important for the patient’s prognosis [18,19].

## 4. First-Line Therapy: Reduced-Intensity Regimens

The issue of therapy toxicity in older cHL patients is so important that there have been multiple attempts over the last 20 years to create the so-called low-intensity treatment regimens, including ChLVPP (chlorambucil, vinblastine, procarbazine, and prednisolone) [20], VEPEM-B (vinblastine, cyclophosphamide, procarbazine, etoposide, mitoxantrone, bleomycin, and prednisolone) [21,22], P-VAG (prednisone, vinblastine, doxorubicin, and gemcitabine) [23], and others. These regimens do not start from the ABVD backbone but are alternative polychemotherapy combinations, some including an anthracycline, while others do not. These regimens have almost always been the subject of small, prospective phase 2 studies whose aim was to find an adequate cycle and then ideally be able to compare the regimen with ABVD. A rare example of a randomized clinical trial compared ABVD and VEPEMB in 54 older patients with cHL (17 localized and 37 advanced); in this selected patient population, it was possible to observe in patients a superiority of ABVD over VEPEMB that, while not statistically significant, was certainly clinically significant, with a 5-year progression-free survival (PFS) of 70% versus 48% (*p* = 0.06) [24]. All these low-intensity regimens aimed to (1) be less toxic and therefore better tolerated, (2) be completed while maintaining an adequate dose rate, (3) lead to a high rate of overall and complete responses, and (4) lead to an improvement in overall (OS) and disease-free survival (DFS).

However, while reduced acute toxicity and a high rate of completion of chemotherapy protocols with adequate dose rates have almost always been observed, the high overall response rates (ORRs) and complete response rates (CRRs) observed did not translate into an improvement in survival curves due to the high recurrence rates seen. Ultimately, less intensity means less toxicity, but it also seems to mean less cure, at least in the setting of older cHL patients.

## 5. First-Line Therapy: Is There a Reference Treatment?

A second strategy is to work on the ABVD backbone to reduce its most frequent toxicities. An interesting attempt in this sense is the one published by Salvi F et al., who replaced the standard doxorubicin in the ABVD scheme with a non-pegylated liposomal formulation, known for its lower incidence of acute and late toxicity (especially as hypokinetic heart disease) in other histologies. In this experience, 47 older and/or cardiac patients were treated. Although neither OS nor PFS was greater than the known rates for these indicators (3-year OS 70%, 3-year PFS 43%), the authors documented how a “standard” ABVD-like therapy in this “protective formulation” was feasible even in patients who would perhaps not have been candidates for anthracycline therapy [25].

Another direction of research is obviously to reduce or omit bleomycin. In the RATHL trial [26], the authors stated that the omission of bleomycin from the ABVD regimen (AVD) after negative findings on interim PET resulted in a lower incidence of pulmonary toxic side effects compared with continued ABVD but not in significantly lower efficacy (3-year PFS 84.4% in the AVD arm versus 85.7% in the ABVD arm). In another study, carried out by the German Hodgkin Study Group (GHSG) [27], the upfront omission of bleomycin from the front-line therapy actually led to a reduction in cure rates, although much smaller than that with the omission of dacarbazine. However, the need to reduce and even eliminate bleomycin toxicity is much greater in older patients. In this setting, in a retrospective series of 147 patients [28], a French group observed how the reduction in or omission of bleomycin (chosen on a clinical basis) did not translate into worse survival rates in the entire population (hazard ratio (HR) for OS: 1.74; 95% confidence interval (CI) 1.0–3.0; *p* = 0.051), while reporting a worse outcome for patients in an advanced stage versus those in a localized stage. A recent Nordic group study [29] retrospectively evaluated registry data from ≥60-year-old patients treated between 2000 and 2021 in Sweden, Norway, and Denmark, who received ABVD (*n* = 671), AVD (*n* = 122), CHOP (*n* = 465), or other regimens (*n* = 296). In this work, no difference in PFS or OS was observed between ABVD and AVD (63% and 64%, respectively, at 5 years), although patients who received AVD were older than those treated with ABVD (74 vs. 66 years).

We can therefore conclude that, at least in the setting of older patients, AVD is a preferable treatment.

Using AVD as a reference and having already discussed the inferiority of attempts to devise low-intensity cycles, we can evaluate which modified AVD cycles are currently available so as to improve AVD efficacy.

## 6. “AVD Plus” Chemotherapy: Attempts to Improve Efficacy

The greatest advance in the treatment of cHL in recent years has undoubtedly been the optimization of first-line therapy with the addition of brentuximab vedotin (BV) to AVD in the ECHELON-1 study [30]. In this phase 3 study, patients with advanced newly diagnosed cHL were 1:1 randomized to receive ABVD or BV + AVD. Of the entire study population, 186 (14%) patients were aged ≥60 years (median age 67 years, range 60–83); an analysis of this subgroup was published in 2022 [31]. With regard to toxicity, the majority of these patients were given a dose reduction or modification of both BV (80% of patients in the BV + AVD arm) and bleomycin (71% of patients in the ABVD arm). The most relevant findings were the high treatment-related mortality in both groups (3.6% BV + AVD vs. 5.1% with ABVD) and the high rate of febrile neutropenia (37% BV + AVD vs. 17% ABVD). The authors reported that the high incidence of febrile neutropenia decreased after the mandatory introduction of primary prophylaxis with G-CSF. Eighteen percent of the patients in the BV + AVD arm experienced grade ≥3 peripheral polyneuropathy but also a substantial reduction in pulmonary toxicity (2% versus 13% in the ABVD arm). In terms of efficacy, 5-year PFS was 67.1% in the BV + AVD group versus 61.6% in the ABVD group (*p* = 0.443). Based on these findings on the older population included in ECHELON-1, BV + AVD can be proposed as an effective bleomycin-free alternative for these patients.

The combination of BV and AVD was also proposed as a sequential therapy (BV 1.8 mg/kg every 21 days for 2 cycles, followed by AVD for 6 cycles, followed by BV 1.8 mg/kg every 21 days for 4 cycles) in a phase 2 study on 48 consecutive newly diagnosed older cHL patients [32]. In this study, the combined sequential modality obtained an ORR of 95% (with CRR of 93%) and a PFS at 2 years of 84%, with low toxicity (4% grade ≥3 peripheral polyneuropathy and 8% febrile neutropenia). Although not investigated further in subsequent studies, a sequential modality such as that proposed by Evens et al. represents a potentially good way forward for the care of older patients [32].

Although the main side effects of the combination of BV + AVD are apparently lower in sequential combination therapy, they should not be overlooked. Along with febrile neutropenia, which can be effectively prevented with G-CSF primary prophylaxis and adequately treated with early empiric antibiotic therapy, grade 3 peripheral polyneuropathy is also an important side effect. Although it is often transient and frequently resolves or improves over time, it can result in a reduction in the ability to perform common activities of daily living (ADLs) [33] and must therefore be appropriately assessed in older patients.

Furthermore, despite the most modern therapies, some factors specific to older patients, such as the presence of comorbidities, continue to play an important prognostic role in terms of overall survival [32], probably due to their influence on chemotherapy-related toxicity and on the possibility of achieving adequate dose intensity. It is therefore clear that correct patient selection must be addressed.

## 7. The Need for Patient Selection and the Role of Simplified Geriatric Assessment

Adequate patient selection for treatments with different intensities or intent should be pursued for a number of reasons, including the possible toxicity of “standard” first-line treatments (as discussed above), the growing incidence of different types of frailty in the older population, and the availability of new effective therapies even for patients not eligible for standard therapy.

There is no univocal definition of frailty in the literature, much less in the setting of patients suffering from hematological cancers, especially in the specific case of patients suffering from cHL. Nevertheless, the literature does demonstrate that frailty, no matter how it is defined, increases as a person ages. The data of Fogg et al. [34], part of a large national study on over 2 million patients in England, showed that frailty (measured with a multiparametric electronic frailty score) was found in 10% of patients aged 50–64 years but in 43.7% of those over age 64 years, reaching very high percentages in the older segments of the population.

Along with fragility, it is also very important to perform an accurate assessment of a patient’s life expectancy. The life expectancy of an older patient with lymphoma is the number of years that separates that patient from the target age that he or she would reach without lymphoma or if the lymphoma can be adequately treated. For a correct estimate of life expectancy, using the reference tables of many Departments of Health, which are updated yearly, and/or online calculators, which are based on these tables, is suggested [35]. By way of an example, in 2023, an 80-year-old woman newly diagnosed with cHL has a life expectancy, in the absence of lymphoma, of about 9.6 more years.

Finally, in the specific setting of cHL, it has been demonstrated that full-dose first-line therapy can produce the best survival results in patients who can tolerate it, resulting in a reduction in mortality from all causes [4]. Indeed, Orellana-Noia et al. observed that receiving conventional therapy had a survival advantage over receiving alternative therapy [19].

In light of the above, it is clear that the accurate assessments of present frailty and life expectancy allow clinicians to distinguish between those patients who will benefit from full-dose treatment and those who will benefit from other treatment options.

A method to stratify patients based on their frailty was developed and tested by the Fondazione Italiana Linfomi (FIL) group, who recently published their results on the usefulness of a simplified geriatric assessment (sGA) in patients with DLBCL. In the FIL study, a baseline assessment of ADL [36], instrumental ADL (IADL) [37], and Cumulative Illness Rating Scale—Geriatric (CIRS-G) [38] made it possible to divide patients into three functional status groups (FIT, UNFIT, and FRAIL), with different outcomes (3-year OS of 75%, 58%, and 43% for FIT, UNFIT, and FRAIL, respectively) [39] (Table 1). Moreover, very similar to what was found in a study conducted by Isaksen et al. [40], the FIL group showed that it was possible to use the stratification of a patient’s functional status for therapeutic indications as well. For example, in patients defined as UNFIT, it was observed that there were no differences in terms of outcome between patients treated with full-dose R-CHOP therapy and those treated with the same therapy at reduced doses (i.e., R-mini-CHOP) [40,41]. The usefulness of such a tool is also related to its practicality: It takes less than 10 min to perform sGA, even in an outpatient setting. The applicability and usefulness of the sGA have yet to be validated in the setting of cHL in older adults; to this end, a prospective study is currently being conducted by the FIL group [42].

## 8. New Ways to Improve Defining Patients’ Functional Status: Sarcopenia and Immunosenescence

Beyond the extremely useful, practical tools to stratify patients in terms of their fitness, it is increasingly evident that functional status is a complex concept involving multiple factors.

Sarcopenia (defined as a mainly cancer-related reduction in muscle mass, strength, and performance) has been widely demonstrated to be a reproducible, effective indicator of the outcome and, to some extent, the tolerance to therapy of patients of any age undergoing chemotherapy in the oncological setting [43,44,45]. More recently, sarcopenia, particularly as a CT-scan measured reduction in muscle mass, was demonstrated to be useful in predicting the outcome of patients with lymphomas as well [46,47,48,49,50,51].

Immunosenescence—an age-related decrease in immune function—is emerging as another important aspect related to lymphoma prognosis and cure. This complex biological process occurs in both the innate and adaptive components of the immune system and results in increased sensitivity to infections, increased autoimmune disorders, reduced immune surveillance, and cancer development [52,53]. Little is known about any possible correlation between immunosenescence and frailty, but it is worth mentioning that in the FIL study published by Tucci et al. [54], the non-FIT DLBCL patients did not benefit from potentially life-saving therapies: The OS of patients treated with a curative regimen was the same as that for those treated with a palliative regimen (2-year OS 19.8% vs. 26.1% for patients treated with curative or with palliative intent, respectively; *p* = 0.85). We could argue that, at least in this study, non-FIT patients may have intrinsic refractoriness to the disease rather than a poor tolerance to treatment, which could be due to the reduced immune surveillance of the tumor. Immunosenescence plays an essential but not entirely understood role in the development of lymphoma, and further studies are needed to better define it.

## 9. Alternative First-Line Therapies (Mainly) for Non-FIT Patients

At this point, a reasonable approach to treatment layering based on the functional status of patients could and should foresee the following factors:

- For FIT patients, a standard chemotherapy regimen (similar to that for younger patients);
- For UNFIT patients, the same standard chemotherapy but with careful monitoring for toxicities and broader use of prophylaxis, or a reduced-dose chemotherapy pathway (not yet validated);
- FRAIL patients represent the most challenging group, as no standard treatment for them currently exists. However, we must bear in mind that numerous treatment schemes have been published in recent years that offer potential alternative therapies for patients who are not candidates for standard chemotherapy.

### 9.1. Brentuximab Vedotin

One type of therapy is based on the use of BV alone or in combination. BV monotherapy as a first-line treatment in older cHL patients has been shown to achieve a good ORR and CRR (92% and 73%, respectively), although of short duration, with a median duration of response and PFS of around 10 months [55]. The good efficacy and low toxicity of BV have led to its evaluation in combination with monochemotherapy, in particular with bendamustine and dacarbazine. BV + bendamustine has proved extremely effective in achieving high ORR and CRR (100% and 88%, respectively) and has very good disease control over time, with a median PFS not reached after a follow-up of approximately 1 year. However, a total of 65% of the patients examined experienced adverse events, and 10% died of treatment-induced toxicity, resulting in the discontinuation of the BV + bendamustine arm of the study [56]. In the same study by Friedberg et al. [56], the combination of BV and dacarbazine proved to be less toxic but adequately effective: for the 22 treated patients, the ORR was 100% (CRR 62%), with a median PFS of 17.9 months (not reached in patients obtaining CR versus 10.8 months in patients without CR) [56].

### 9.2. Anti-PD1-Containing Therapies

Anti-PD1-containing therapies represent a great opportunity, especially given their extreme efficacy in cHL (greater than that observed in any other histology of oncological disease) and their good toxicity profile, making them an extremely interesting pharmacological class, especially for older patients.

Considering their use as first-line therapy in this population, anti-PD1-containing combinations have been primarily studied in order to improve the efficacy of standard treatments: the combination of nivolumab with AVD in the phase 2 CheckMate 205 study obtained an 84% ORR (CRR 67%) in a cohort of 51 patients, with 9-month PFS of 92% and 9-month OS of 98% [57].

The use of anti-PD1-containing therapy alone or in combination with other drugs allows clinicians to offer appropriate treatment to those patients who are not candidates for standard therapy and who therefore may not be offered treatment at all. In this context, the combination of BV + nivolumab has proved to be well tolerated and effective. Although the ACCRU trial [58] discontinued its enrollment after the interim analysis because it had not achieved the primary CRR objective (at the final evaluation of 46 patients, the authors observed an ORR of 61% and a CRR of 48%), it illustrated that the responses obtained were long-lasting (median PFS 18.3 months for the entire population, in particular, not reached for patients with CR, versus 6 months for patients with PR, with a median follow-up of 21.2 months). The efficacy of this combination was also demonstrated by Yasenchak et al. [59], who documented an ORR of 95% (CRR 79%) in their 19 patients, with a median PFS not reached at a median follow-up of 19.4 months.

The French NIVINHO trial on 56 patients consisted of a first phase of treatment with nivolumab monotherapy (240 mg flat dose) every 14 days for 6 administrations and subsequent continuation based on response: The patients in complete metabolic response continued nivolumab monotherapy for an additional 18 cycles, while those in partial response or stable disease were treated with a combination of nivolumab and vinblastine every 14 days for 18 cycles. Thanks to this scheme, this difficult-to-treat population (median age 75 years, median CIRS-G 10) achieved an ORR of 46.5% and a CRR of 28.6% (16% post-nivolumab in monotherapy), with a median PFS of 9.8 months at a median follow-up of over 20 months [60].

Finally, several study protocols are about to be activated (e.g., the GHSG HD20 study “Indie trial” NCT04837859 and the announced GHSG HD19 and UK RATIFY trials) in which anti-PD1-containing therapies will be used to reduce the toxicity or duration of first-line therapy and, in some cases, to allow the omission of chemotherapy in patients in complete metabolic response.

## 10. What Second-Line Options Are Available and Effective?

Salvage therapy in relapsed/refractory (R/R) older cHL patients is even more complex since the issues encountered in first-line therapy naturally increase in a salvage setting due to the presence of a clearly more aggressive disease and to the patient’s prior treatment regimens (including in terms of toxicity). It is also evident that the problem of salvage therapy in a patient population in which the diminished efficacy of first-line therapies is well known is even larger because of the greater frequency of patients with R/R disease. A well-developed study by the GHSG [61] a few years ago revealed an advantage, in terms of response to second-line therapy and overall survival, for “low-risk” patients who were treated with polychemotherapy schemes compared with patients who were candidates for an intensification procedure (autologous transplantation) or palliative therapy. The authors described a simple prognostic score for recurrence based on the presence of advanced disease, anemia, and early relapse: Patients with no or only one risk factor had a 3-year OS of 59%, whereas patients with two or all three risk factors had a 3-year OS of only 9%. At present, the results of this work encounter some application limitations: In particular, the clinical trend of patients in general (both on the first line and on the subsequent lines) and the prognostic value of the identified score are strongly affected by the new therapeutic scenario. On the one hand, the advent of new drugs (especially BV and anti-PD1) has decidedly increased the possibility of saving even older patients. On the other hand, the increasingly early use of these drugs, even in first-line therapy, is radically changing the treatment paradigm of patients with cHL.

Finally, an adequate assessment of the patient’s functional status could be useful in the setting of second-line therapy as well; more intensive and potentially effective approaches with low toxicity could be offered to FIT patients, reserving more conservative approaches to patients with the most compromised functional status (e.g., BV or pembrolizumab monotherapy). The possible advantage of an adequate patient selection is demonstrated by the fact that in a small series of 15 highly selected older patients aged 60–67 years (median age 64 years), an autologous stem cell transplant (ASCT) with alternative conditioning regimen (etoposide 60 mg/kg i.v. over 8 h on day-4, melphalan 180 mg/m^2^ i.v. over 30 min on day-3) proved to be safe and effective, with no transplant-related deaths and with a 3-year PFS and OS of 73% and 88%, respectively [62]. In a French retrospective study by Stamatoullas et al. [63] on 128 FIT patients undergoing ASCT with BEAM conditioning, ASCT showed low toxicity and achieved good disease control over time (5-year PFS and OS of 54% and 67%, respectively). The GELTAMO group analyzed a retrospective series of 121 patients aged 50 years or older who underwent ASCT [64], including 42 patients aged ≥60 years. The authors were able to demonstrate very good disease control, with a PFS and OS at 10 years of 51% and 57%, respectively, without any substantial differences between younger and older patients. Moreover, in the multivariable analysis, excluding pre-transplant disease status, the only factor associated with an unfavorable outcome was comorbidities, not age. In all these investigations, the correct stratification of patients’ functional status emerged as a real need, with an evident clinical impact, allowing most FIT patients to start intensifying programs that allow them to obtain high disease-free and global survival rates. Currently, however, no comprehensive GA validation studies are available in this setting.

Most older patients with R/R cHL are not eligible for an ASCT intensification strategy. Frequently, gemcitabine-based or bendamustine-based approaches have been used as salvage treatments [65,66,67], but the most valid therapeutic alternatives are based on the use of new drugs. BV monotherapy can achieve OR and CR rates of 56% and 38%, respectively, although disease control over time is not optimal, with a median PFS of 9 months (18.5 months for CR patients) [68]. Anti-PD1-containing therapies are also good alternatives, but once again, the studies on the older population are extremely few. An interesting exception is the KEYNOTE-204 study, which randomized 300 patients with R/R cHL to receive BV or pembrolizumab. Of these patients, 49 aged ≥65 years achieved a PFS of 8.2 months with pembrolizumab versus 5.5 months with BV [69]. Unfortunately, no data regarding the functional status of these older patients were collected, and the number of patients was too small to draw definitive conclusions.

Currently, patients with R/R elderly cHL who are not eligible for intensification strategies are in most cases candidates for treatment programs with more containment than curative purposes. In this situation, a case-by-case discussion of the treatment program with the patient, including not only the expected benefits but also the foreseeable side effects, is fundamental. Therapeutic alternatives with similar efficacy but lower costs must also be considered. A subset of low-risk patients, unfit for ASCT, may be salvaged with radiotherapy [61], which can also be widely and safely used in the setting of elderly cHL treatment as a post-chemotherapy consolidation strategy or as a palliative approach.

## 11. Conclusions

cHL in older adults remains challenging because of both its greater biological aggressiveness and the unsatisfactory response to first-line therapy. AVD can be considered the gold standard in these older patients, and attempts to improve its efficacy with the addition of BV (especially as sequential therapy) seem to translate into better outcomes. Adequate patient stratification at baseline by employing sGA tools that include ADL, IADL, and comorbidity scores remains essential. FIT patients may be considered for treatment approaches similar to those for younger patients, while new effective, more suitable therapies for UNFIT and FRAIL patients are emerging, such as the combinations of BV with dacarbazine or nivolumab. There is a growing need for prospective studies to better characterize these patients, to validate an sGA that includes emerging factors such as sarcopenia and immunosenescence, and to investigate new therapies both in the first-line and salvage settings, with particular focus on the role that new agents can play.

## Figures and Tables

**Table 1:** FIL criteria for sGA assessment.

Criteria	FIT	UNFIT		FRAIL
ADL	≥5 ^a^	<5 ^a^	6 ^a^	<6 ^a^
IADL	≥6 ^a^	<6 ^a^	8 ^a^	<8 ^a^
CIRS-G	0 score = 3–4, ≤8 score = 2	≥1 score = 3–4, >8 score = 2	0 score = 3–4, <5 score = 2	≥1 score = 3–4, ≥5 score = 2
Age	<80	<80	≥80	≥80

Abbreviations: ADL, activity of daily living; CIRS-G, Cumulative Illness Rating Scale—Geriatrics; IADL, instrumental ADL; sGA, simplified geriatric assessment. ^a^ Number of residual functions.
